# Increase in driving after cocaine use in Spain: a cross-sectional dataset analysis for 2021

**DOI:** 10.3389/fpubh.2023.1178300

**Published:** 2023-05-09

**Authors:** Mercedes García-Mingo, Marta Martín-Fernández, Eduardo Gutiérrez-Abejón, F. Javier Álvarez

**Affiliations:** ^1^Institute for Alcohol and Drug Studies, Pharmacology and Therapeutics, Faculty of Medicine, University of Valladolid, Valladolid, Spain; ^2^Pharmacological Big Data Laboratory, Pharmacology and Therapeutics, Faculty of Medicine, University of Valladolid, Valladolid, Spain; ^3^Centro de Investigación Biomédica en Red de Enfermedades Infecciosas (CIBERINFEC), Instituto de Salud Carlos III, Madrid, Spain; ^4^Pharmacy Directorate, Castilla y León Health Council, Valladolid, Spain; ^5^CEIm, Hospital Clínico Universitario de Valladolid, Valladolid, Spain

**Keywords:** alcohol, cannabis, breath alcohol concentration, cocaine, driving under the influence, oral fluid, psychotropic drugs, street drug testing

## Abstract

**Objective:**

Driving under the influence of alcohol and/or drugs impairs skills essential for safe driving, increases the risk of being involved in a traffic accident and is particularly prevalent in Spain. The aim is to assess the prevalence of positive substance driving cases, what factors may be associated with driving after substance use, and the evolution of the progress in the prevalence of drug use among drivers in drivers based on the 2008, 2013, 2018, and 2021 studies.

**Study design and setting:**

The present study was conducted in a representative sample of Spanish drivers in 2021 for alcohol (breath) and psychoactive substances [oral fluid (OF)]. The sample size was 2980 drivers, mostly males (76.5%) with a mean age of 41.35 ± 13.34 years.

**Results:**

In 2021, 9.3% of drivers tested positive for alcohol and/or drugs. The presence of alcohol alone was observed in 4.2% of drivers, alcohol and another substance in 0.3%, a single drug in 4.4%, and two or drugs other than alcohol in 0.4%. Overall, cocaine cases were the highest registered in 2021 (2.4%), while cannabis (1.9%) and polydrug cases (0.7%) were the lowest, with respect to the 2008/2013/2018 studies.

**Conclusions:**

According to our research, in 2021, 9 out of 100 drivers were detected to have some substance in their system. This prevalence remains unacceptably high in Spain, with a marked increase in the frequency of driving after cocaine use. Further interventions and measures must be taken to avoid driving under the influence of alcohol and/or drugs.

## 1. Introduction

The use of alcohol, illicit drugs, and some medicines can impair certain skills necessary for safe driving and increase the likelihood of a traffic collision (1–3). Driving after drug use is a major problem that hampers efforts to improve road safety (4). Laws regarding driving after consuming alcohol and/or drugs may vary from country to country. However, the implementation of new traffic policies and interventions to prevent driving after consuming alcohol and/or drugs should be a worldwide focus (2, 3).

The prevalence of drug use in Spain is one of the highest among developed countries, especially for cocaine and cannabis (5). It is well known that a large proportion of alcohol and/or drug users actually drive after using substances (6, 7). Thus, previous studies have shown that the prevalence of positive cases for alcohol and/or drugs is higher in Spain than in most European Union countries (3, 8).

The current legislation in Spain (9) establishes the prohibition of driving with the presence of drugs in the driver's body, except for substances used under medical prescription and for therapeutic purposes. Roadside drug testing of oral fluid was regulated in 2010 (10). Meanwhile, the zero-tolerance legal system was subsequently established in 2014 (9, 11, 12). Drivers are penalized €1,000 has 6 points taken off their license if any number of substances other than alcohol are found in their system or if they refuse to take the drug screening test. Driving with a blood alcohol level >0.5 g/l (>0.25 mg/l on a breath test) is punishable by law. However, the application of the limits for novice and professional drivers are more restrictive [>0.3 g/L (0.15)]. In addition, a pictogram on the packaging of medicines in Spain informs patients and physicians of the risk of using driving-impairing medicines (13).

In 2020, 48,194 roadside drug tests were performed in Spain, of which 35.1% were positive: 32,124 were performed in routine roadside checks, of which 34.2% were positive for any illicit drug. In the case of drivers who had committed an infraction, the percentage increased to 47.3% (*n* = 9,697) and when it was the result of an accident, the percentage decreased to 21.3% (*n* = 6,373) (14).

As part of the European Driving Under the Influence of Drugs, Alcohol and Medicines (DRUID) project, a study was carried out on the presence of drugs in drivers. Spain participated in this study in 2008 (3, 8). Further studies using a very similar methodology were conducted in 2013 (15), in 2015 (16, 17), 2018 (11, 12, 14), and now in 2021.

The present study is conducted in a representative sample of Spanish in drivers in 2021 for alcohol (breath) and psychoactive substances [oral fluid (OF)], with the aim of: (1) analyze the prevalence of positive results, (2) to assess what factors may be associated with driving after substance use, and (3) to evaluate the progress in the prevalence of drug use among drivers bases on the 2008, 2013, 2018 and 2021 studies.

## 2. Materials and methods

The study design and methodology are consistent with previous studies conducted in 2008, 2013, and 2018 (7, 10, 11, 14).

### 2.1. Design

The study was conceived by the Spanish National Traffic Agency by recruiting drivers of motor vehicles circulating on Spanish public roadways during 2021. A total of 3,009 drivers were tested. However, 28 tests could not be completed due to device malfunction (1 alcohol test) or insufficient saliva for testing (27 drug tests).

Drivers were randomly recruited through 128 checkpoints set up in 4 geographical areas (Cantabrian, Mediterranean, North, and South). The stratification of the sample was based on the population size, road type (urban/interurban), and time period: “*weekday and time of day: a) Monday to Friday from 7:00 to 23:59 (weekday); b) Monday to Friday from 0:00 to 6:59 (weekday early morning); c) Saturday, Sunday, and holidays from 7:00 to 23:59 (weekend day); and d) Saturday, Sunday, and holidays from 0:00 to 6:59 (weekend early morning)*”(11). All drivers were tested for alcohol and drugs.

Table 1 shows the sociodemographic characteristics of the drivers participating in the 2021 study. Sampling was conducted over a 4-week period from October 8 through November 4, 2021. Ethical approval was obtained (CEIm Área de Salud Valladolid Este, reference PI-21-2461, dated October 28, 2021).

**Table 1:** Sociodemographic characteristics of drivers participating in the 2021 study.

		**Total 2,881**	
		***n***	**%**
**Gender**	Males	2,281	76.5
	Females	633	21.3
	No answer/do not know	66	2.2
**Age groups (years)**	16–24	313	10.5
	25–34	757	25.39
	35–44	696	23.35
	45–49	387	13.00
	50 or more	807	27.09
	No answer/do not know	19	0.65
**Nationality**	Spain	2,468	82.8
	Latin America	113	3.8
	Morocco	65	2.2
	Other countries	49	1.6
	Other European countries	51	1.7
	European Union	127	4.3
	No answer/do not know	108	3.6
**Type of vehicle**	Motorized Bike	28	0.9
	Motorcycle	230	7.7
	Other	287	9.6
	Private car	2,411	80.9
	Non-registered vehicles	25	0.8
**Area**	Cantabrian	405	13.6
	Mediterranean	803	27
	North	547	18.4
	South	1,225	41.1
**Type of road**	Interurban	1,452	48.7
	Urban	1,529	51.3

An anonymized dataset was constructed that included sociodemographic information (sex, age, nationality) as well as date, location, road type, vehicle type, driver's license type, breath alcohol test results (in mg/L), and oral fluid drug test results (in ng/ml). These data were organized into 6 general groups: alcohol, cannabis, cocaine, opiates, amphetamines + their analogs, and benzodiazepines + their analogs.

The primary categorization of the variables was positive or negative for each substance. Results were then categorized as (1) positive for alcohol (without other drugs), (2) positive for alcohol and drug(s), (3) positive for two or more drugs (without alcohol), and (4) positive for a single drug (without alcohol). For cases positive for a single drug, the type of drug was recorded (cannabis, cocaine, opiates, amphetamine + analogs, or benzodiazepine + analogs; Supplementary Table 1).

### 2.2. Drug and alcohol test procedure

Mandatory roadside alcohol and drug testing was conducted by Traffic Police officers. Alcohol testing was conducted by breath test and drug testing was conducted by OF. In addition, roadside rapid detection kits were used for drug testing (cannabis, cocaine, amphetamine, methamphetamine, and opioids).

Breath alcohol testing was performed using the Dräger Alcotest^®^ 6810, 6820, 7410, 7110, 7510, and Envitec AlcoQuant^®^ 6020 devices. Results were reported as mg of alcohol per liter of exhaled air. Results higher than 0.05 mg/L were consideredpositive. Positive results were then stratified into three ranges: 0.051–0.15 mg/L, 0.16–0.25 mg/L, and 0.26 or more mg/L. Additionally, drug testing was performed using the SoToxa™ Mobile Test System, Dräger DrugTest^®^ 5000 or Alere™ DDS^®^ 2 Mobile Test System.

For substances other than alcohol, positive cases were confirmed and quantified by an accredited toxicology laboratory (SynLab, accredited laboratory according to ISO17025 norm). This required the collection of a second OF sample of ~1 mL. Positive drug tests were confirmed by liquid chromatography coupled to tandem mass spectrometry (LC-MS/MS). The analytical results of the drugs were provided by the Spanish National Traffic Agency. Only confirmatory drug analysis results were used in the current study.

Laboratory-tested substances and their limits for confirmatory analysis are shown in Supplementary Table 2. For medications, results were considered positive if they were above the specified cut-off (Supplementary Table 2). In this sense, information on prescription or non-prescription by a physician was not available.

### 2.3. Statistical analysis

SPSS V.26.0 (Statistical Package for Social Sciences) was used for statistical analysis. Percentages with their 95% confidence intervals (95%CI) were used to quantify positive tests. Mean ± standard deviation (SD) was used to report the age of the drivers. The chi-square test (χ^2^) was used to determine differences between categorical variables. A backward step-wise multivariate logistic regression model was applied to determine whether age, sex, road type (urban/interurban), time of week (weekday/night or weekend day/night), vehicle type, and region of the country (Cantabria, North, Mediterranean, or South) had any effect on testing positive. A two-tailed z-test was performed to analyze results between the 2021, 2018, 2013, and 2008 studies. The linear relationship between positive cases for any substance, cannabis and cocaine was performed with respect to the study years (2008, 2013, 2018, and 2021). A statistical significance level of *p* ≤ 0.05 was established.

## 3. Results

Of the 2,980 drivers included in the study, 76.5% were male, with a mean age of 41.35 ± 13.67, ranging in age from 16 to 88 years. Drivers between the ages of 35 and 49 were strongly represented (36.4%). Spanish nationality was predominant (82.8%). Most drivers driving a passenger vehicle (80.9%), and driving on urban roads (51.3%) (Table 1).

Alcohol and/or drug test results were positive in 9.3% of drivers included in the study (Table 2). Alcohol alone was detected in 4.2% of cases, alcohol in addition to another substance in 0.3% of cases, a single drug in 4.4% of cases, and more than one class of drug different from alcohol in 0.4% of cases. About half of the drivers who tested positive for alcohol had concentrations ≥ 26 mg/L. Supplementary Table 3 shows the results in relation to sex: as a trend, driving after substance use tended to be more frequent among males, although the difference was not always statistically significant. Supplementary Table 4 shows the results in relation to the 5-year age range.

**Table 2:** Prevalence of cases testing positive for alcohol and/or drugs in Spanish motor vehicle drivers.

	**2008** **(****7****)**		**2013** **(****14****)**		**2018** **(****10****)**		**2021**				
	***n***	**% (95% CI)**	***n***	**% (95% CI)**	***n***	**% (95% CI)**	***n***	**% (95% CI)**	**2008 vs. 2021 p** ^*^	**2013 vs. 2021 p** ^*^	**2018 vs. 2021 p** ^*^
**No substance**	2,838	86 (84.7–87.1)	2,658	90.7 (89.7–91.8)	2,566	90.7 (89.7–91.8)	2,703	90.7 (89.6–91.7)			
**Substance**	464	14.1 (12.9–15.3)	274	9.3 (8.2–10.3)	315	10.9 (9.8–12.1)	277	9.3 (8.3–10.4)	**0.0001**	0.947	0.037
**Alcohol alone** **>** **0.05 mg/ml**	162	4.9 (4.2–5.7)	100	3.4 (2.3–4.1)	111	3.4 (2.3–4.1)	125	4.2 (3.5–5)	0.177	0.115	0.506
**Alcohol** **+** **drugs**	56	1.7 (1.3–2.1)	22	0.7 (0.4–1)	25	0.7 (0.4–1)	10	0.3 (0.2–0.6)	**0.0001**	**0.030**	**0.008**
**Several drugs**	18	0.6 (0.3–0.8)	26	0.9 (0.6–1.2)	31	0.9 (0.6–1.2)	11	0.4 (0.2–0.6)	0.304	**0.012**	**0.001**
**Only one drug**	228	6.9 (6–7.8)	126	4.3 (3.6–5)	148	4.3 (3.6–5)	131	4.4 (3.7–5.2)	**0.0001**	0.853	0.183
**Cannabis**	174	5.3 (4.5–6)	92	3.1 (2.5–3.8)	108	3.1 (2.5–3.8)	55	1.9 (1.4–2.4)	**0.0001**	**0.001**	**0.0001**
**Cocaine**	42	1.3 (0.9–1.7)	26	0.9 (0.5–1.2)	30	0.9 (0.5–1.2)	72	2.4 (1.9–3)	**0.001**	**0.0001**	**0.0001**
**Amphetamine and analogs**	2	0.1 (0–0.2)	4	0.1 (0.0–0.3)	2	0.1 (0–0.3)	2	0.1 (0–0.2)	0.918	0.403	0.973
**Opiates**	5	0.1 (0–0.3)	1	0 (0.00–0.1)	6	0 (0–0.1)	2	0.1 (0–0.2)	0.317	0.573	0.143
**Benzodiazepine and analogs**	5	0.2 (0–0.3)	3	0.1 (0–0.2)	2	0.1 (0–0.2)	0	(0.0)	–	–	–

95% CI, confidence interval. ^*^Two tailed two proportions Z-test.

Table 3 shows the results of the multivariate logistic regression analysis. These results indicate that the likelihood of a driver's sample being positive for some substance was related to male sex [Odds Ratio (OR), OR = 1.7], decreased with increasing age of the driver (OR = 0.97), and occurred more frequently during the weekend-night period (OR = 2.5) and the weekday-night period (OR = 1.5), and in the south area (OR = 1.6).

**Table 3:** Logistic regression analysis: statistically significant variables in cases testing positive for alcohol and/or drugs in Spanish motor vehicle drivers, 2021.

	**Any substance**		**Alcohol alone**		**Alcohol** + **Drugs**		**Only one Drug (no alcohol)**		**Several Drugs (no alcohol)**	
	**% (95% CI)**	***p***	**% (95% CI)**	***p***	**% (95% CI)**	***p***	**% (95% CI)**	***p***	**% (95% CI)**	***p***
**Age (years)**	**0.97 (0.96–0.98)**	**0.0001**					**0.96 (0.95–0.97)**	0.0001	**0.96 (0.94–0.99)**	**0.0001**
**Gender** Reference: female	**1.7 (1.3–2.3)**	0.0001	2.2 (1.2–3.7)	0.007					**2.9 (1.3–6.3)**	**0.006**
**Period of the week**										
**Reference: weekdays**										
**Weekend nights** 0.00–6.59	**2.5 (1.9–3.4)**	0.0001	6.5 (4.0–10.6)	0.0001						
**Weeknights** 0.00–6.59	**1.5 (1.03–2.1)**	**0.03**	2.7 (1.5–4.8)	0.001						
**Type of vehicle**										
**Reference: other vehicles**										
**Private car**			3.5 (1.7–7.1)	0.001			**0.5 (0.3–0.7)**	0.0001		
**Region of the country**										
**Reference: Cantabria**										
**South**	**OR** **=** **1.6, (1.1–2.3)**	0.01					**2.6 (1.5–4.5)**	0.0001		
**Road type** Reference: urban									**0.5 (0.3–0.8)**	**0.007**

95% CI, confidence interval.

The prevalence of positive cases in 2021 (Table 2, 9.3%, *z* = 5.83, *p* = 0.0001), 2018 (10.9%, *z* = 3.65, *p* = 0.0003), and 2013 (9.3%, *z* = 5.70, *p* < 0.0001) was lower than that reported in 2008 (14.1%). The frequencies of 2021 and 2013 are not different (*z* = 0.066, *p* = 0.947), but the figures of 2021 are lower than those of 2018 (*z* = 2.081, *p* = 0.037). Finally, the 2018 figures are higher than those of 2013 (*z* = 1.96, *p* = 0.05).

**Figure 1:**
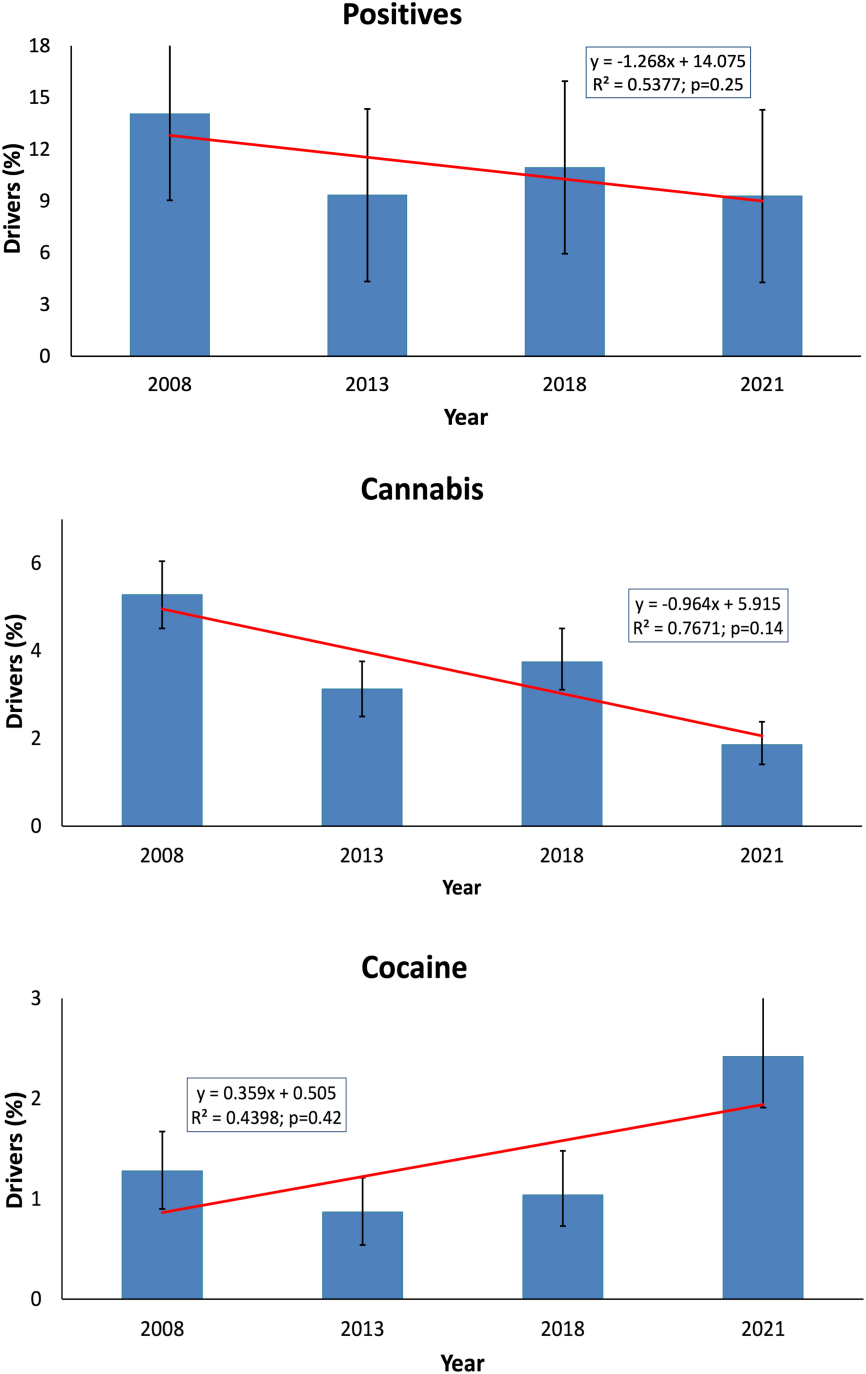
Positive cases to any substance (alcohol and/or drugs), cannabis, and cocaine in the 2008, 2013, 2018, and 2021 roadside studies are presented. Frequency is shown with its 95% confidence interval. Red dotted lines represent the theoretical linear regression line. The equation of the line is displayed with its R2 and *p*-values.

The figures from 2021 to 2018 were lower not only in terms of the prevalence of positive cases for any substance, but also for alcohol + drug positive cases, multiple drug positive cases, and cannabis positive cases. The only increase was observed in the frequency of cocaine-positive cases, which more than doubled from 2018 (1%) to 2021 (2.4%, *z* = −4.024, *p* < 0.0001, Table 2). Overall, cocaine figures for 2021 were the highest registered, while cannabis cases were the lowest. Figure 1 shows the trend over the years for cannabis and cocaine positive cases, although no linear relationship could be established.

## 4. Discussion

According to our findings, the prevalence of positive tests for alcohol and/or drugs remains high in Spain in 2021: nine out of 100 drivers continue to drive after consuming substances. In general, the figures for 2021 were lower compared to 2018. The only increase was in the frequency of cocaine positives, which more than doubled compared to 2018. Overall, cocaine figures for 2021 were the highest recorded across studies, while cannabis figures were the lowest across studies.

Before analyzing the results of alcohol and/or drug consumption in drivers, the prevalence of alcohol and/or drug consumption in the general population should be determined. Therefore, according to a survey conducted by the Ministry of Health (18), in 2022 in Spain 76.4% of the population consumed alcohol, 13.1% consumed hypnotics, 10.6% consumed cannabis and 2.4% consumed cocaine.

In general, the frequency of drug-impaired driving in 2008 (before the introduction of oral fluid drug screening on the road, and a zero-tolerance legislation implementation), is higher than in later studies. Although this study cannot determine whether the introduction of roadside drug testing and new regulations (19) influenced the frequency of driving after drug use, such changes may have contributed to the observed decline (15, 20).

The prevalence of driving under the influence of alcohol and/or drugs in Spain is considerable, above the European average (7.4%) (2, 3, 21, 22). Nevertheless, due to the different methodologies used in the available studies, it is not easy to make comparisons with other countries such as the United States. In this sense, according to the results of a survey, the prevalence of people driving after consuming alcohol and/or drugs in the United States is 7.7% in the population aged 26 and over, rising to 15% in those aged 21 to 25 (23).

Despite these prevalence data, in 2021, the number of road fatalities in Spain decreased to 31.8 victims per million inhabitants, below the European average (45), but still above countries such as Germany (30.9), Ireland (27.4), Scotland (23.9), and Denmark and Switzerland (23.1). Measures such as reducing speed limits, increasing the presence of roadside cameras, introducing more traffic sanctions and restricting the use of electronic devices, especially cell phones, appear to be contributing to this decline (9). Moreover, in countries outside of continental Europe, results vary and are generally higher. For example, the number of road fatalities is around 240 per million inhabitants in Brazil (24), 117 in the United States (25), and 49 in Australia (26).Therefore, these figures make Europe the continent with the highest level of road safety (27).

Looking at the statistics on the presence of alcohol and/or drugs among injured drivers or deaths in traffic collisions, the results are overwhelming. Alcohol is undoubtedly the most common impairing driving substance in road fatalities, accounting for 20–25% in Europe (28, 29), 20–30% in Australia (30), and 28% in the United States (31). It is noteworthy that in most studies, alcohol is often combined with other substances, especially cocaine and cannabinoids (32).

In addition to alcohol, the use of illicit drugs must be considered. In Europe, the prevalence of drug use among injured drivers or deaths in traffic collisions is 14–17% (5), while in the United States it is estimated to be 16% (33). The type of substance most used varies according to the geographical area of the driver, with cocaine predominant in southern European countries (16, 34–36), amphetamines in northern European countries (37) and cannabinoids in countries such as New Zealand (38), the United States (23) and France (5).

An important fact in the 2021 study is the decrease in polydrug use, which is 0.7% in 2021, lower than in previous studies: 2018 (1.9%), 2013 (1.6%) and 2008 (2.2%). The risk of suffering a traffic collision has been demonstrated to be strongly associated with polydrug use (2, 3, 39). Preceding studies, particularly on fatalities, have shown that polydrug use is common (22), as have data from roadside drug testing in Spain between 2011 and 2016 (40, 41). Therefore, the implementation of new traffic policies aimed at the population, both drivers and pedestrians, to raise awareness of the risks of driving after consuming alcohol and/or drugs is a priority.

Figures for the year 2021 confirmed that men are more likely to drive after substance(s) abuse than women, as previously described (42), decreases with driver age, and is more likely to occur at night, either on weekends or weekdays. In terms of driver sex, men are more likely to be behind the wheel in Spain (57 vs. 43%) (43). On the other hand, as in previous studies (8, 11, 12, 15) the percentage of positive cases (alcohol or drugs) for non-alcoholic drugs, cannabis and cocaine is particularly high in southern Spain.

As noted above, cocaine use among drivers doubled in 2021, which is concerning. In our study, cocaine was detected in 2.4% of drivers, which is consistent with the prevalence of cocaine use in the general Spanish population (18). Cocaine is known to impair driving ability (2). The risk of driving under the influence of cocaine is similar to driving with a blood alcohol concentration between 0.5 g/L to 0.8 g/L (RR = 2–10) (3). On the other hand, the relative risk of fatality after cocaine use is 2.96 (95% CI 1.18–7.38) (2). This risk increases exponentially when cocaine use is combined with other substances, especially alcohol (3, 39, 44).

Limitations of this study and the 2008, 2013, and 2018 studies of drug prevalence among drivers have been described previously (8, 11, 15, 16). Although the 4 studies used the same methodology, some differences may have influenced the results. First, the road tests were conducted at 4 different times in 2008, at 2 different times in 2013, and during a single 4-week period in 2018 and 2021. Second, the way in which traffic density was determined at the roadside checkpoints was different in each study. In addition changes in the prevalence of use of different substances over the years may have influenced the differences between the results of the 4 studies. All studies included the same substances and used the same cut-off levels. Finally, a limitation is the lack of data on the use of the so-called “*new psychoactive substances*”, such as synthetic cannabinoids, cathinones, ketamine, gamma-hydroxybutyrate (GHB), m-chlorophenylpiperazine (*mCPP*), and substances of plant origin (khat, kratom), among others.

## 5. Conclusion

In conclusion, our figures show that in 2021, 9% of Spanish drivers consumed some substance behind the wheel. This prevalence is still unacceptably high. However, changes in Spanish traffic laws in 2010 and 2014, as well as an increase in the number of roadside checkpoints for drug testing, have contributed to a decrease in the presence of alcohol and/or drugs in drivers compared to data from previous studies. In this work, an increase in the frequency of driving after cocaine use was noted, while a decrease in driving after cannabis and polydrug use was observed. In general, driving under the influence of alcohol and/or drugs remains particularly prevalent in Spain, and further interventions and measures are needed to prevent driving under the influence of alcohol/drugs. Measures such as increasing the number of traffic checks, increasing economic sanctions, and raising driver awareness should be the main topics for the authorities in creating new traffic policies.

## Data availability statement

The raw data supporting the conclusions of this article will be made available by the authors, with the prior approval of the Dirección General de Tráfico.

## Ethics statement

The studies involving human participants were reviewed and approved by the CEIm Área de Salud Valladolid Este, reference PI-21-2461, dated October 28, 2021. Written informed consent for participation was not required for this study in accordance with the national legislation and the institutional requirements.

## Author contributions

Conceptualization: MM-F, EG-A, and FÁ. Data curation, investigation, and software: MM-F, EG-A, and MG-M. Formal analysis: EG-A, MG-M, and FÁ. Funding acquisition, project administration, resources, supervision, and visualization: FÁ. Investigation: MM-F, EG-A, and MG-M. Methodology: MG-M and EG-A. Validation: EG-A and FÁ. Writing – original draft: MG-M and FÁ. Writing – review and editing: MM-F, EG-A, MG-M, and FÁ. All authors contributed to the article and approved the submitted version.
