# Supplementary material for: Increase in driving after cocaine use in Spain: a cross-sectional dataset analysis for 2021
Source: Front Public Health. 2023 May 9;11:1178300. doi: 10.3389/fpubh.2023.1178300 (PMC10203456; doi:10.3389/fpubh.2023.1178300)
Supplement: Supplementary file 1 [file Data_Sheet_1.pdf]

Supplementary Material

1 Supplementary Figures and Tables

1.1 Supplementary Tables

Supplementary Table 1. Substance categories (3).

**Substance name or group of substances**	**Substance(s) included in each group**
Alcohol	Alcohol
Cannabis	Cannabis
Opiates	6-Acetylmorphine, Codeine, Morphine, Methadone
Cocaine	Cocaine, benzoylecgonine
Amphetamine and analogues	Amphetamine, Methamphetamine, MDA, MDMA, MDEA
Benzodiazepine and analogues	7-Aminoclonazepam, Alprazolam, Clonazepam, Diazepam, Flunitrazepam, Lorazepam, Nordiazepam, Oxazepam, 7-Aminoflunitrazepam<br><br>Zolpidem, Zopiclone

Supplementary Table 2. Substances and sample cut-off concentrations for laboratory confirmation according to DRUID guidelines (3).

Substance	Cut-off concentration oral fluid (ng/mL)
6-Acetylmorphine	16.0
7-Aminoclonazepam	3.1
7-Aminoflunitrazepam	1.0
Alprazolam	3.5
Amphetamine	360.0
Benzoylecgonine	95.0
Clonazepam	1.7
Cocaine	170.0
Codeine	94.0
Diazepam	5.0
Flunitrazepam	1.0
Lorazepam	1.1
MDA	220.0
MDEA	270.0
MDMA	270.0
Methadone	22.0
Methamphetamine	410.0
Morphine	95.0
Nordiazepam	1.1
Oxazepam	13.0
THC	27.0
Tramadol	480.0
Zolpidem	10.0
Zopiclone	25.0

Supplementary Table 3. Prevalence of cases testing positive for alcohol and/or drugs among Spanish drivers, by gender.

	Male		Female		χ2	p
	n	% (95% CI)	n	% (95% CI)		
No substance	2043	89.6 (88.3-90.8)	600	94.7 (92.9-96.3)		
Any substance	238	10.4 (9.2-11.7)	34	5.3 (3.8-7.3)	15.081	p<0.0001
Alcohol alone >0.05mg/ml	109	4.8 (4-5.7)	14	2.2 (1.3-3.6)	8.076	p=0.004
Alcohol + drugs	8	0.3 (0.2-0.7)	2	0.3 (0.1-1)	0.018	p=0.895
Several drugs	10	0.4 (0.2-0.8)	1	0.2 (0-0.7)	1.036	P=0.309
Only one drug	112	4.9 (4.1-5.9)	16	2.6 (1.5-4)	6.697	p=0.010
Cannabis	43	2 (1.5-2.6)	12	2 (1.1-3.3)	0.004	p=0.947
Cocaine	65	3 (2.4-3.8)	4	0.7 (0.2-1.5)	11.009	p=0.001
Amphetamine and analogues	2	0.1 (0-0.3)	0	0.0	0.570	p=0.450
Opiates	2	0.1 (0-0.3)	0	0.0	0.570	p=0.450
Benzodiazepine and analogues	0	0.0	0	0.0		

frontiers

Supplementary Table 4. Prevalence of cases testing positive for alcohol and/or drugs among Spanish drivers by age.

	Any substance		Alcohol and drug		Drugs (no alcohol)		Alcohol alone > 0,05mg/ml	
**Age groups**	**n**	**% (95% CI)**	**n**	**% (95% CI)**	**n**	**% (95% CI)**	**n**	**% (95% CI)**
16-20	8	9.3 (4.4-16.6)	0	0.0	5	5.4 (2.2-12.1)	3	4 (1-8.9)
21-25	29	9.7 (6.7-13.4)	1	0.2 (0-1.6)	12	4 (2.2-6.7)	16	5.5 (3.2-8.3)
26-30	66	16.5 (13.1-20.4)	3	0.8 (0.2-2)	34	8.4 (6.1-11.6)	29	7.2 (5-10.1)
31-35	33	9 (6.4-12.3)	1	0.3 (0-1.3)	20	5.6 (3.5-8.2)	12	3.2 (1.8-5.5)
36-40	34	11.3 (8-15.1)	1	0.2 (0-1.5)	24	7.9 (5.3-11.3)	10	3.2 (1.7-5.8)
41-45	42	10.1 (7.4-13.2)	1	0.2 (0-1.1)	29	6.9 (4.8-9.7)	12	2.9 (1.6-4.8)
46-50	17	5 (3-7.6)	1	0.2 (0-1.4)	7	1.9 (0.9-3.9)	10	2.9 (1.5-5.1)
51-55	19	8 (4.9-11.7)	0	0.1 (0-1)	5	2.2 (0.8-4.5)	14	5.7 (3.3-9.2)
56-60	10	4.6 (2.3-7.8)	2	0.9 (0.2-2.9)	5	2.5 (0.9-4.9)	3	1.2 (0.4-3.6)
>= 61	16	5.8 (3.5-8.9)	0	0.0	1	0.5 (0-1.7)	15	5.3 (3.2-8.5)
χ^2, p	45.265, p<0.0001		6.397, p=0.700		48.592, p<0.0001		22.039, p=0.009	
